# Manual restrictions on Palaeolithic technological behaviours

**DOI:** 10.7717/peerj.5399

**Published:** 2018-08-16

**Authors:** Alastair J.M. Key, Christopher J. Dunmore

**Affiliations:** School of Anthropology and Conservation, University of Kent, Canterbury, Kent, United Kingdom

**Keywords:** Manual Dexterity, Manipulation, Force, Oldowan—Acheulean transition, Stone tool innovation, Flint knapping

## Abstract

The causes of technological innovation in the Palaeolithic archaeological record are central to understanding Plio-Pleistocene hominin behaviour and temporal trends in artefact variation. Palaeolithic archaeologists frequently investigate the Oldowan-Acheulean transition and technological developments during the subsequent million years of the Acheulean technocomplex. Here, we approach the question of why innovative stone tool production techniques occur in the Lower Palaeolithic archaeological record from an experimental biomechanical and evolutionary perspective. Nine experienced flintknappers reproduced Oldowan flake tools, ‘early Acheulean’ handaxes, and ‘late Acheulean’ handaxes while pressure data were collected from their non-dominant (core-holding) hands. For each flake removal or platform preparation event performed, the percussor used, the stage of reduction, the core securing technique utilised, and the relative success of flake removals were recorded. Results indicate that more heavily reduced, intensively shaped handaxes with greater volumetric controls do not necessarily require significantly greater manual pressure than Oldowan flake tools or earlier ‘rougher’ handaxe forms. Platform preparation events do, however, require significantly greater pressure relative to either soft or hard hammer flake detachments. No significant relationships were identified between flaking success and pressure variation. Our results suggest that the preparation of flake platforms, a technological behaviour associated with the production of late Acheulean handaxes, could plausibly have been restricted prior to the emergence of more forceful precision-manipulative capabilities than those required for earlier lithic technologies.

## Introduction

The production and use of flaked stone tools were likely important to the survival of Palaeolithic hominins. The potential influence of these manually demanding behaviours on the evolution of the human hand has long been recognised (Napier, 1962; Marzke, 1983; Marzke, 1997; Marzke, 2013; Williams, Gordon & Richmond, 2010; Rolian, Lieberman & Zermeno, 2011; Key & Lycett, 2011; Kivell, 2015; Almécija & Sheerwood, 2017; although see Almécija & Alba, 2014). Recent research has also demonstrated how the manual anatomy and associated biomechanical capabilities of different hominin species may have influenced the nature of the Palaeolithic archaeological record (Marzke & Shackley, 1986; Rolian, Lieberman & Zermeno, 2011; Domalain, Bertin & Daver, 2017; Key et al., 2017; Patiño et al., 2017; Key & Lycett, in press). That is, the types, forms and technological strategies of stone tool artefacts may have been limited by, or preferentially selected for, as a result of how effectively hominins could use the hand when manipulating or securing lithic objects. Research concerning how the evolution of the hominin hand may have been influenced by stone tool production and use has been reviewed in detail elsewhere (Marzke, 1997; Marzke, 2013; Kivell, 2015; Almécija & Sheerwood, 2017). The present article reciprocally focuses on the influence that hominin manual capabilities may have had on the types and forms of stone tools produced during the Lower Palaeolithic.

The earliest intentionally flaked stone tools are currently from the 3.3 million-year-old site of Lomekwi 3, West Turkana (Kenya), and appear to have been directed towards producing large flake cutting tools through passive-hammer or bipolar percussive strategies (Harmand et al., 2015; Lewis & Harmand, 2016). Subsequent to ∼2.6 million years ago (Mya) Oldowan flake and core technologies, typically characterised by the expedient production of variably sized flake cutting tools from hand-held cores using hard-hammer percussion, appear more widely across East Africa (Kimbel et al., 1996; Roche et al., 1999; Semaw et al., 2003; Rogers & Semaw, 2009; Hovers, 2012; Reti, 2016; Proffitt, 2018). Simple flake and core stone tools are thereafter ubiquitous throughout the Palaeolithic. After ∼1.75 Mya large bifacially flaked core tools (‘bifaces’) appear in the archaeological record across East Africa as part of the Acheulean techno-complex (Lepre et al., 2011; De la Torre & Mora, 2014; Diez-Martín et al., 2015). These early Acheulean tools, often characterised by handaxes and cleavers, are typically thought to have been produced using hard-hammer percussion. Bifaces go on to typify the next >1 million years of the archaeological record across the Old World (Lycett & Gowlett, 2008; Gowlett, 2015; Moncel et al., 2015) until the onset of Middle Palaeolithic technologies ∼300 Kya (Moncel et al., 2011; Tryon & Faith, 2013; Adler et al., 2014). The nature and extent of any chronological changes to stone technology during the Acheulean are debated (e.g., Vaughan, 2001; Chauhan, 2009; Gowlett, 2011; McNabb & Cole, 2015; Moncel et al., 2015; Gallotti, 2016), however, there are indications that later Acheulean bifacial tools (handaxes in particular) were at times produced using soft-hammer percussion, became thinner relative to their width (more ‘refined’), displayed greater evidence of intentional thinning, volume control (mass distribution), investment (e.g., time, skill), shaping and symmetry (Gowlett, 1986; Saragusti et al., 1998; Schick & Clark, 2003; Grosman, Goldsmith & Smilansky, 2011; Beyene et al., 2012; García-Medrano et al., 2014; Li et al., 2018; Moncel et al., 2016; Gallotti & Mussi, 2017; Iovita et al., 2017; Shimelmitz et al., 2017), and at times displayed evidence of platform preparation prior to a flake’s removal (Stout et al., 2014). Together, these technologies describe ∼3 million years of stone tool production and use during the Lower Palaeolithic.

Relationships between technological or morphological aspects of Lower Palaeolithic stone tools and hominin manual capabilities are often mentioned, but rarely tested, in archaeological literature (e.g., Crompton & Gowlett, 1993; Delagnes & Roche, 2005; Machin, 2009; Lycett & Von Cramon-Taubadel, 2015). Although paleoanthropologists frequently debate whether fossil hominin hand anatomy could facilitate stone tool related precision grips, it is rarely the case that specific technological or morphological aspects of these tools are discussed (although see Tocheri et al. (2008) for an example). Therefore, there are only a few instances where hypothesised relationships between technological or morphological features of Lower Palaeolithic stone tools and hominin manual capabilities have actually been investigated.

Regarding the origin of the first flaked stone tools, Rolian, Lieberman & Zermeno (2011) used a metal ‘simulated flake tool’ to calculate the external moments, internal flexion moments and joint stresses of tool users. Their data suggested that efficient flake tool use with low biomechanical stresses may not have been possible prior to the evolution of the derived pollical anatomy observed in later *Homo* (Rolian, Lieberman & Zermeno, 2011). Recently, Key & Lycett (in press) demonstrated the significant impact that tool user biometric variation can have on stone tool-use efficiency across the Lower Palaeolithic, revealing that relationships between biometric parameters and tool-use efficiency depend on the type of tool being used and the biometric variable under consideration. Their results suggest that the effective use of flakes and handaxes is not only dependent on hominins displaying relatively strong hands, but that the onset of Acheulean handaxes may have been linked to the evolution of more anatomically modern manual dimensions (Key & Lycett, in press). Williams-Hatala et al.’s (2018) investigation of manual pressure variation during flake and handaxe use may also indicate there to be differences in grip loading levels dependent on the size of the tool gripped.

These results are, in part, due to the variable grips required when securing different Lower Palaeolithic tools, as described by Marzke & Shackley (1986). Manual demands and grip choices have also been demonstrated to vary during different stone tool production sequences (Marzke & Shackley, 1986). Comparisons between flake and handaxe production, for example, identified differences in the motion of the dominant arm, with the latter requiring smaller, more precise flaking actions. The authors also suggest that a ‘lighter grip’ could be used to secure an Oldowan flake core relative to a handaxe or pick when detaching flakes (Marzke & Shackley, 1986). As cores become smaller over a reduction sequence, Marzke and colleagues (Marzke & Shackley, 1986; Marzke et al., 1998) describe how the distal aspects of digits are increasing heavily recruited and the palm is used less. An early experiment also suggested that lower thumb to finger length ratios may have precluded early hominin’s ability to firmly secure handaxes during production, in turn resulting in “very crude handaxes” during the early Acheulean (Krantz, 1960: 116). Key et al. (2017) found that experienced knappers gripped hammerstones with high pressure when detaching particularly large flakes. In turn, large stone flakes within Lower Palaeolithic archaeological sequences (Sharon, 2010; Shipton et al., 2014) plausibly indicate that hominins were capable of exerting and resisting high manual pressures during precision (hammerstone) manipulation.

Aside from manual requirements, other studies emphasise the increased cognitive demands of handaxe production relative to Oldowan flakes (Stout et al., 2008; Muller, Clarkson & Shipton, 2017), while Mateos, Terradillos-Bernal & Rodriguez (in press) have recently experimentally compared the energetic cost of soft and hard hammer handaxe production. Only Faisal et al. (2010), however, have empirically examined Lower Palaeolithic technological transitions from a manipulative perspective. Joint angles on, and abduction angles between, the digits of the non-dominant hand of a skilled flint knapper indicated that, at least for the individual under investigation, Acheulean and Oldowan stone tool production are “indistinguishable” in terms of manipulative complexity (Faisal et al., 2010: 6). Faisal et al.’s (2010) study also highlights key manipulative differences between these two reduction sequences, including the unique need to properly and securely brace a handaxe as it becomes increasing thin relative to its width.

Together, these studies emphasise the distinct manual demands required by the type and form of stone tool being used or produced. These demands must be facilitated by effective grips, which are, in turn, facilitated by anatomical adaptations. Without this anatomy it is unlikely that the respective tool forms would be found in associated archaeological deposits. Yet, there is still relatively little known about hand recruitment during the production of different types and forms of stone tool. Further, there is limited information about the effect biomechanical variation in a tool producer’s hand has on the efficacy of different stone tool production behaviours. Certainly, the onset and adoption of certain technological or morphological features in the Palaeolithic archaeological record could have been restricted by biomechanical capabilities, including the forceful precision grip capabilities of the hominin upper limb.

The non-dominant hand is known to experience high loading levels and perform complex manipulative tasks during the production of stone tools (Marzke & Shackley, 1986; Faisal et al., 2010; Key & Dunmore, 2015), perhaps to a greater extent than the dominant hand. Differences in manipulative requirements between stone tool production behaviours might, then, be more readily detected in this hand relative to the dominant hand. Here, we test the null hypothesis that the pressures experienced across the non-dominant hand of stone tool producers during a series of Lower Palaeolithic technological activities, including a range of tool types produced and percussors used, are not significantly different. Further, we assess how flake removal success is related to the pressure used to secure cores and whether manual pressures vary according to the stage of a core’s reduction, or the technique used to support a core against hammerstone impact reaction forces.

## Methods

### Reduction strategies and technological differences

**Figure 1:**
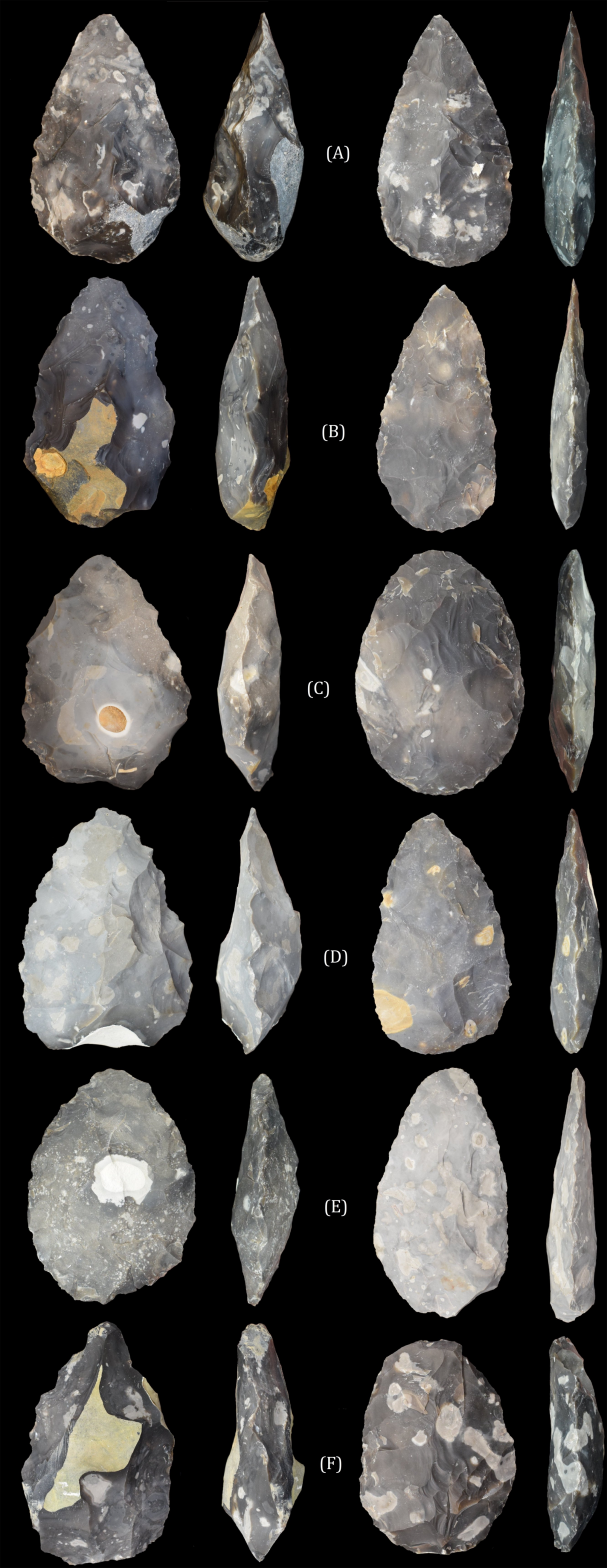
The ‘early Acheulean’ and ‘late Acheulean’ handaxes produced by knappers 6 (A), 9 (B), 5 (C), 4 (D), 3 (E) and 2 (F). Handaxes are not presented to scale in order to emphasise shape differences. Source: A Key.

**Figure 2:**
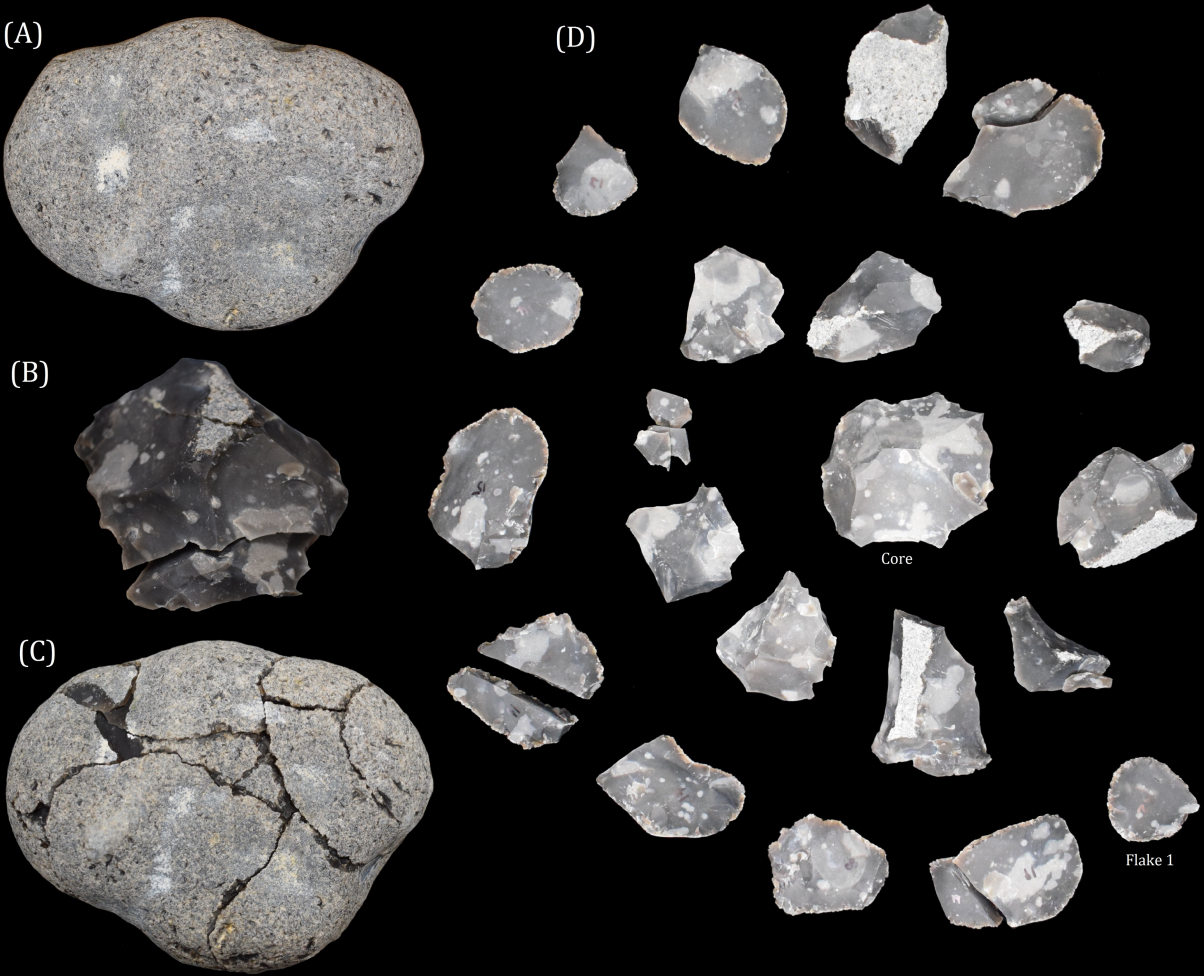
A replica Oldowan flake core knapped by the lead author. The original unmodified core (A), a flake after its removal from the core at a late stage of the reduction (B), and the refitted core (C) are depicted on the left. The sequence of flake removals can be seen on the right (D). The first flake removed is highlighted on the bottom right hand side of the image, with subsequent flake removals spiralling clockwise into the centre and ending with the core. Source: A Key.

Three Lower Palaeolithic reduction strategies are examined here: (1) the production of replica Oldowan flake tools (‘flake’), (2) bifacial flake removals while shaping an ‘Early Acheulean’ handaxe (EAH), and (3) bifacial flake removals while shaping a ‘Late Acheulean’ handaxe (LAH) (Figs. 1 and 2). Both flake and EAH tools were produced via hard hammer percussion while LAH were produced with soft hammer percussion as well. The latter strategy also employed specialist grinding stones during the preparation of flake platforms. The terms EAH and LAH used here refer to general increases in flaking extent, shaping, volume control, symmetry, the use of intentional ‘thinning’ flakes, soft-hammer percussion and prepared flake platforms in later Acheulean handaxes (Saragusti et al., 1998; Schick & Clark, 2003; Grosman, Goldsmith & Smilansky, 2011; Diez-Martín et al., 2014; Stout et al., 2014; Gallotti & Mussi, 2017; Iovita et al., 2017; Shimelmitz et al., 2017). While these differences are often clearest when tools produced >1 Mya are compared to those produced after ∼0.5 Mya, we do not mean to imply uniform linear progression of forms across regional records (Vaughan, 2001; Gowlett, 2013; Moncel et al., 2015; McNabb & Cole, 2015). Rather, we seek to investigate if handaxe forms produced using distinct techniques may be limited by biomechanical capabilities, as inferred from manual pressure records (see below).

Although the translation and rotation of cores are manually demanding behaviours (Marzke et al., 1998; Key & Dunmore, 2015), the present analysis focuses only on manual pressure while securing cores during flake removals or platform preparation activities (edge grinding, retouching and trimming). As these behaviours remove mass from a core, they shape a lithic artefact and have the potential to be identified from the archaeological record.

**Figure 3:**
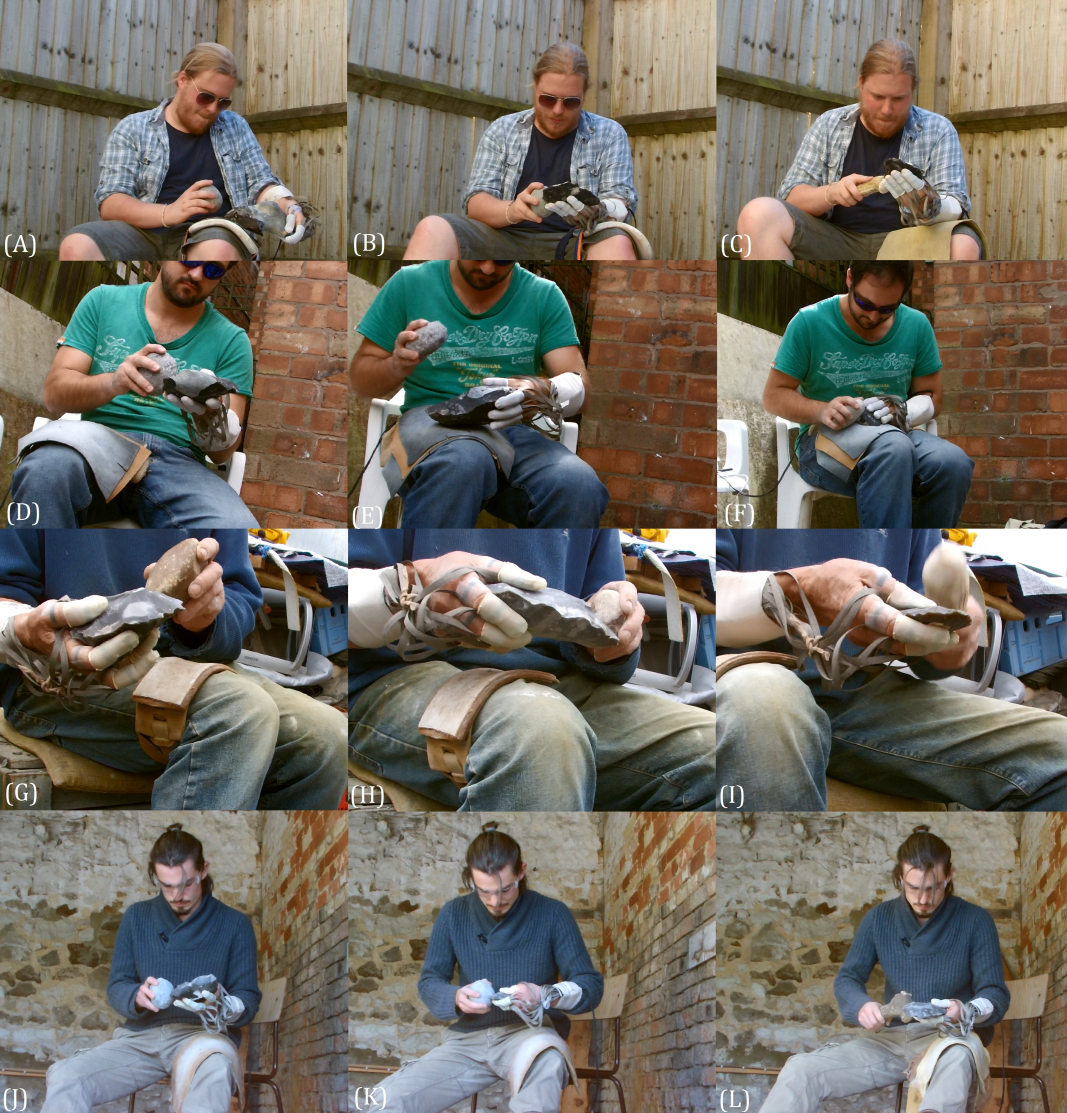
Four of the knappers during the three reduction sequences. Oldowan flake and core (A, D, G, J), early Acheulean handaxe (B, E, H, K) and late Acheulean handaxe (C, F, I, L) reduction sequences are depicted. Note image F, where the knapper is grinding an edge in preparation for a flake removal. Images used with permission. Source: A Key.

Nine skilled flint knappers, each with at least five years experience, took part in the study. At a minimum, all individuals were capable of consistently producing replica Acheulean handaxes of predetermined form when required. Notably, some of the participants exceeded this lower skill threshold by a considerable margin (*cf.* Eren et al., 2014). All had previously knapped while connected to manual pressure sensors and are familiar with producing tools within other experimental conditions (Winton, 2005; Williams, Gordon & Richmond, 2010; Key & Dunmore, 2015; Key et al., 2017). Additionally, most knap on a professional and frequent basis (e.g., academic, craftsman etc.) and likely provide the best possible sample available for providing natural, unfettered, pressure data. For these reasons, we are confident in the use of a single trial per reduction strategy for each knapper (collected within a single day) and the repeatability of the data collected. Each individual undertook the flake reduction first, followed by the EAH and then LAH sequence (Fig. 3). British flint from Suffolk and Kent was used in all reductions. All tool production sequences were recorded using a HD video camera. Ethical approval was granted by the School of Anthropology and Conservation Ethics Committee (University of Kent; Ref. Ares 19065). All individuals gave informed consent.

**Figure 4:**
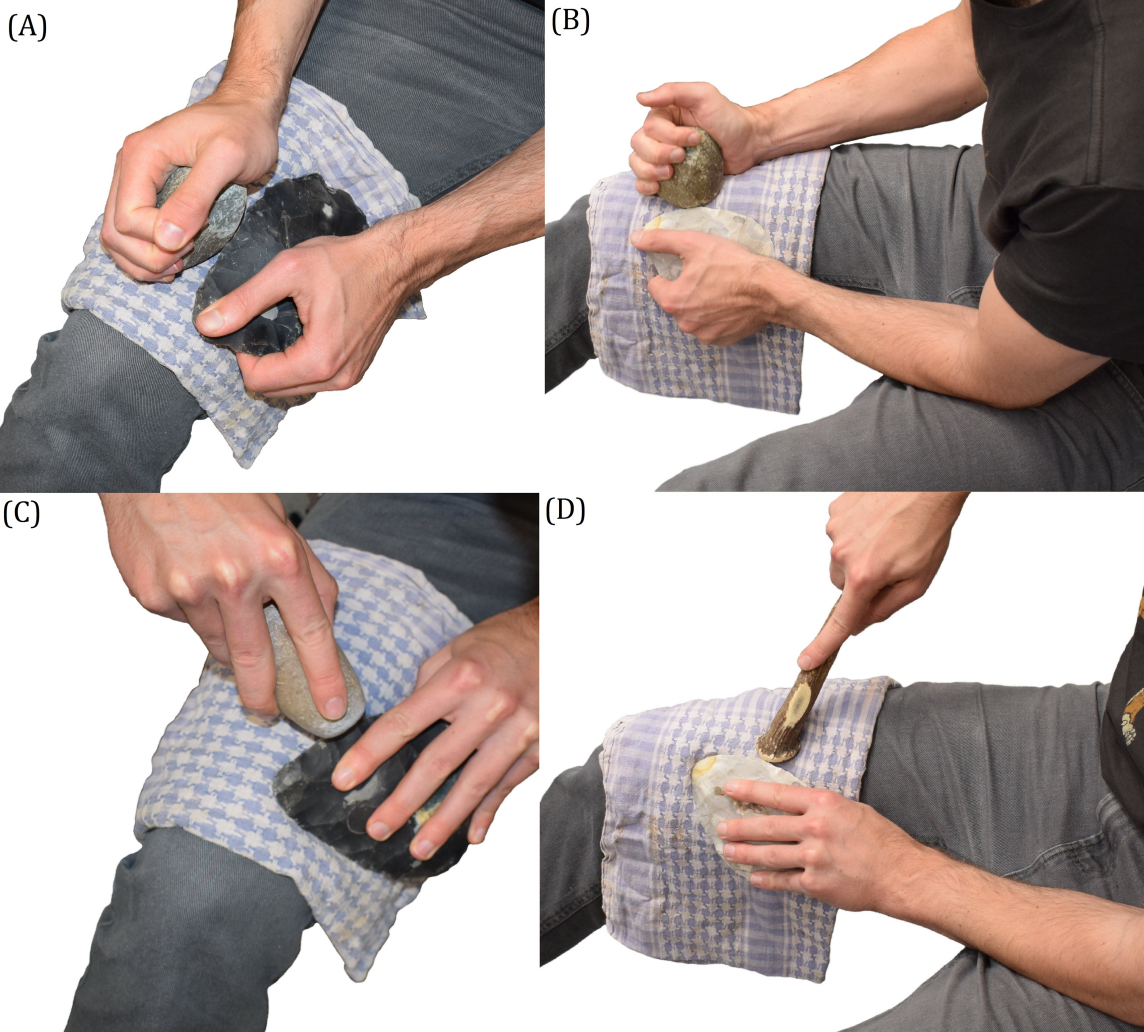
Platform preparation events. Two edge grinding (A, B) and retouching (C, D) events are depicted. These behaviours are undertaken to remove edge ‘lipping’ (i.e., peaks at the apex of an edge that prevent a direct blow to the intended point of impact), to round the edge, to alter the flake platform’s angle or to shape and isolate platforms. Source: A Key.

Each knapper used their own hammerstones and soft hammers, without restriction, although red deer (*Cervus eleghus*) and moose (*Alces alces*) billets were typically used. No wooden or copper billets were used. Knappers were free to use grinding stones during platform preparation events in the LAH reduction, although in many instances soft and hard hammers were also used for grinding and trimming (Figs. 3 and 4). Knappers produced flakes at their own pace and supported the core in whatever way they preferred (this varied between the core resting in the hand or on the leg). Every attempted flake removal was coded as successful if the flake detached or unsuccessful if it did not. In instances where a fracture had clearly propagated through the core but required additional minor taps to remove it, the original hammer strike was considered successful and the small taps were not included in the study. Small (micro) flake removals undertaken when preparing platforms for large flake’s removals are considered as distinct to ‘flake removals’ in this study.

### Pressure sensors

**Figure 5:**
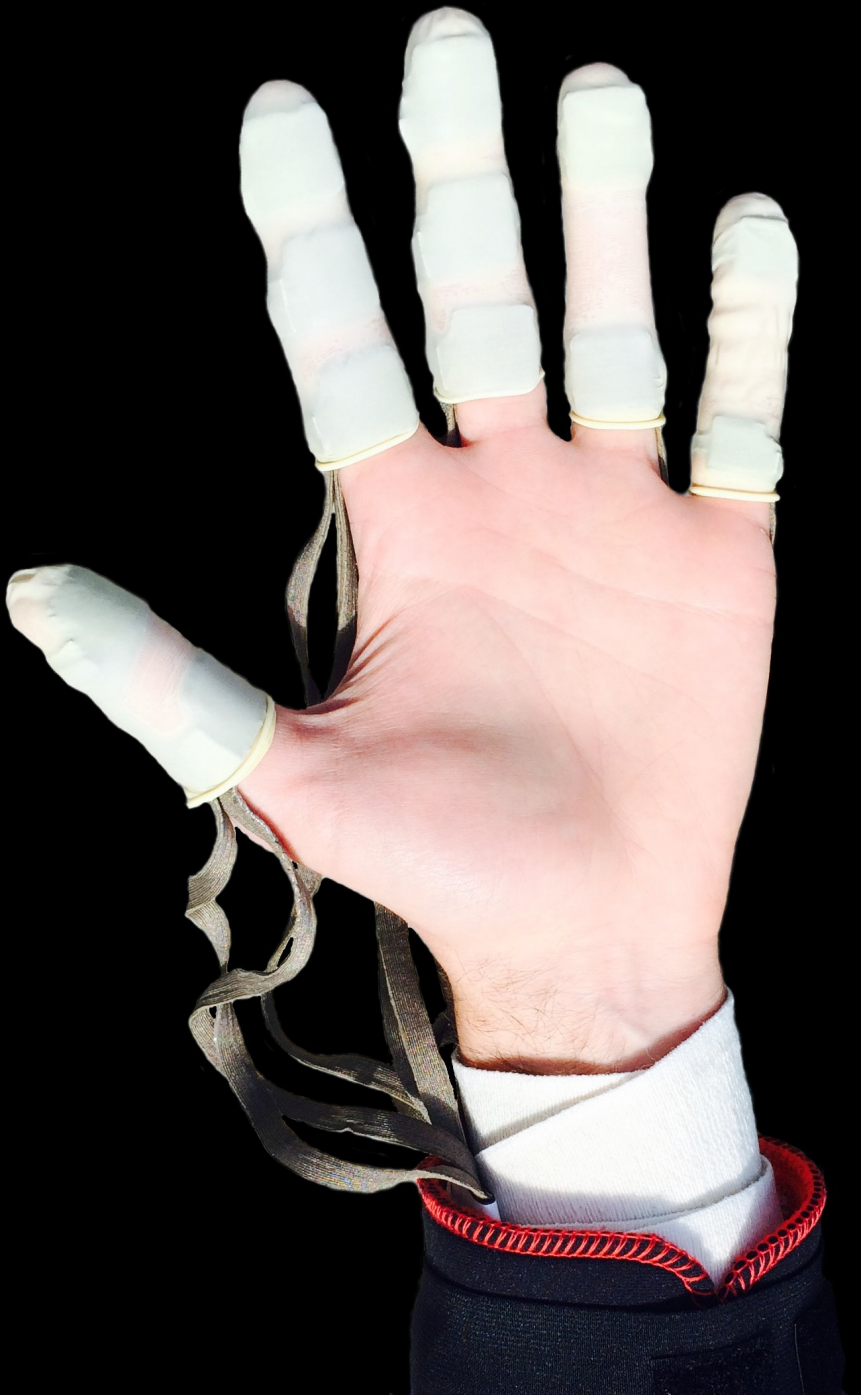
The Novel Pliance^®^ pressure system attached to the hand of knapper #1. Note the 12 sensors secured underneath the finger cots. Source: A Key.

A wireless Novel Pliance^®^ sensor system was used to record the pressures (kPa) experienced across the non-dominant hand of knappers during all three reductions (Fig. 5). The system was comprised of 10 17 ×17 mm^2^ and two 10 ×10 mm^2^ sensors. The larger sensors were attached to the distal and proximal phalanges of digits 1–4 as well as the intermediate phalanges of digits 2 and 3. The two smaller sensors were attached to the distal and proximal phalanges of digit 5 (Fig. 5). All sensors were attached to the palmar surfaces of digits using double-sided tape and Velcro straps. Latex finger cots were used to protect the sensors and help keep them in place. The sensors were ‘zeroed out’ prior to data collection starting to account for any potential pressure caused by the finger cots. In all instances data were collected at a rate of 50 Hz.

### Data extraction

Reduction sequences ranged between 5 to 34 min in duration. The number of individual data points collected from sensors ranged from ∼12,000 to ∼102,000. To identify individual behavioural instances within data streams it was necessary to align the pressure data output with the video records of each reduction sequence. Knappers were asked to free their non-dominant hand of any loads prior the reduction sequence starting and forcefully pinch their thumb and index finger. This created a known behaviour that was clearly identifiable at the start of the pressure data and the video record, after which, the two outputs could be accurately aligned.

**Figure 6:**
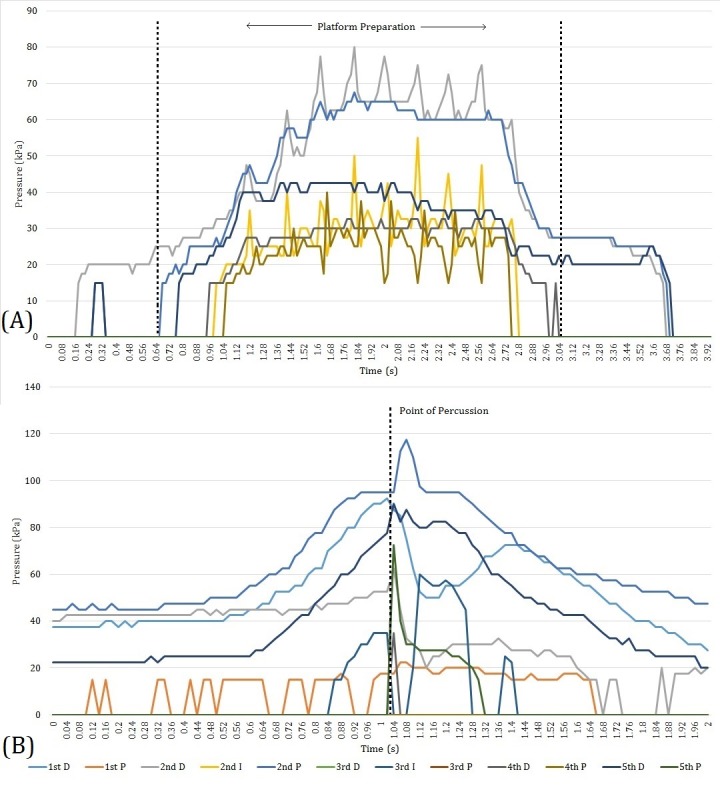
Two data sequences from which peak pressure values were recorded. Depicted in the image (A) is a brief platform preparation event during a LAH sequence. In this instance a grinding event is shown, with the peaks and troughs associated with the forwards and backwards motion of the abrading stone being clearly visible. The image (B) is from a flake removal during the same LAH reduction. It is clear that prior to the point of percussion pressure increases. At the point of impact, however, there is a noticeable peak as sensors record both the pressure exerted by the digits and those in reaction to hammerstone impact forces. Two sensors display a drop in pressure at the point of impact, presumably as the core moves off the sensors in reaction to the impact. ‘D’, ‘I’, and ‘P’ refer to the distal, intermediate and proximal sensor on each digit (respectively).

Every time one of the behaviours under investigation was performed the peak pressure (kPa) experienced on each sensor was identified and recorded. For an attempted flake removal, peak pressures were identified from 2-second-long segments of the data stream (1 second either side of the point of impact; Fig. 6). Platform preparation behaviours could occur for substantially longer periods, therefore peak pressures were extracted from across their entire duration. Every manual activity recorded here, and therefore every peak pressure value, was assigned a technological strategy (flake, EAH, LAH), an indenture type (hard hammer, soft hammer, grinding stone), a removal type (successful flake, unsuccessful flake, platform preparation), a core-support position (leg, hand), and a sequence number.

Pressure data from all 12 sensors were summed to produce a record of the digital peak pressures experienced at a whole-hand level during individual technological behaviours. For each statistical comparison the peak pressures from all nine participants were combined. Participant seven’s distal sensor on the first digit became detached during his flake reduction sequence. To make this discrepancy equal across all conditions examined here, no data for this sensor from this participant were included in the analyses.

To control for inter-knapper differences in pressure, records were normalised to a 0-1 scale by dividing the difference between each peak pressure record and minimum record of that reduction sequence, by the range of values in that reduction. Since all reductions begin in a similar manner this scaling should not preclude the identification of significant differences between groups.

### Statistical analyses

#### Pressure differences between flake, EAH, and LAH reduction strategies

Both successful and unsuccessful flake removal data were used to investigate how pressure varies between the three core reduction strategies. Shapiro–Wilk tests revealed that normalised peak pressure data were not normally distributed in any of the three reduction strategies (*p* ≤ .0001). As reduction sequence lengths varied between knappers, each was sub-sampled to *n* peak pressure records evenly spaced over that sequence length, where *n* was the minimum length of sequence data analysed (*n* = 30) (File S1). This step ensured that knappers that produced longer sequences were not over-represented in the data, while still yielding reasonable statistical power with a sample of 270 peak pressure records in each reduction type. A Friedman test and post-hoc pairwise Wilcoxon signed rank tests were used to test for significant differences between normalized median pressure values between each reduction type. Significant values were identified at *p* < .017 as a Bonferroni correction was applied.

#### Pressure according to flake removal success

Average pressure was compared between flake removals depending on whether they were successful or not, within each reduction strategy. Hard and soft hammer percussion were included in the LAH analyses, but platform preparation events were not. Shapiro–Wilk tests confirmed that all three data sets were not normally distributed (*p* ≤ .040). Mann–Whitney *U* tests were repeated individually for Flake, EAH and LAH reductions as these data were not repeated measures. Significance was assumed in-line with the Bonferroni correction (*p* ≤ .017).

#### Pressure differences between core support strategies

As the present investigation is one of a few to consider core securing events with the non-dominant hand, we also analysed how different core support strategies may influence manual pressures. Two methods of core support were naturally used by knappers during reductions. Cores were either secured and supported solely in the hand, with the palm and fingers working to support their weight, or by the hand bracing tools against the leg. Pressure differences between these two core support strategies were compared individually within the three reduction strategies using Mann–Whitney *U* tests as Shapiro–Wilk tests identified that all data sets were not normally distributed (*p* ≤ .0003). Significant values were identified at *p* < .017 as a Bonferroni correction was applied. The LAH data does include platform preparation events using both core support strategies.

#### Pressure according to mass removal method

Only the LAH reduction displayed multiple mass removal (core shaping) methods; namely, hard and soft hammer flake removals, and platform preparation events. To examine how pressure varies between each of these three mass removal strategies LAH data were separated and then compared by technique used. Shapiro–Wilk tests confirmed that the three data sets were not normally distributed (*p* ≤ .0001). In turn, peak pressures were statistically compared between the three strategies using sub-sampled data as for testing differences between reduction strategies, though here the lowest number of mass removals in a sequence of a given type was 11 and so each removal type pressure sample was constituted of 99 records evenly spaced over reduction sequences (File S1). A Friedman test and post-hoc pairwise Wilcoxon signed rank tests were used to test for significant differences between normalized median pressure values between each mass removal type.

#### Pressure differences dependent on reduction stage

To examine whether core reduction stage significantly influences the pressure exerted and resisted by the non-dominant hand, flake sequence numbers were regressed on summed peak pressure data for each respective reduction type. This analysis of the influence of a core’s stage of reduction, as defined by the number of flakes removed, on manual pressure does not use normalized or sub-setted data since it is the covariance of these variables that is under investigation. Due to the influence that core form, knapping mistakes, raw material inclusions, and participant enthusiasm could have on the duration of tool production sequences, there is potential for later trends within shorter sequences to be concurrent with earlier stages of longer reduction sequences. In turn, if there is only an increase in pressure during the final stages of a handaxe’s production, for example, then this trend in the shorter sequences may go undetected. Hence, we performed another regression using flake removal sequence numbers of equal range that were proportionally normalised to the shortest sequence length (out of the nine) for each reduction type. This allows assessment of manual pressure from the start of a reduction sequence relative to its end (as determined by the tool producer) irrespective of any variation in the number of flake removals.

Both sets of regressions are performed with all nine participants’ data. Regressions were repeated individually for each of the three reduction strategies. Only hard and soft hammer flake removals were included in these first analyses for the LAH data. Pressure data from platform preparation event sequences were independently investigated using both types of regression. Significance was assumed in-line with the Bonferroni correction (*p* ≤ .0125) in each instance.

## Results

**Figure 7:**
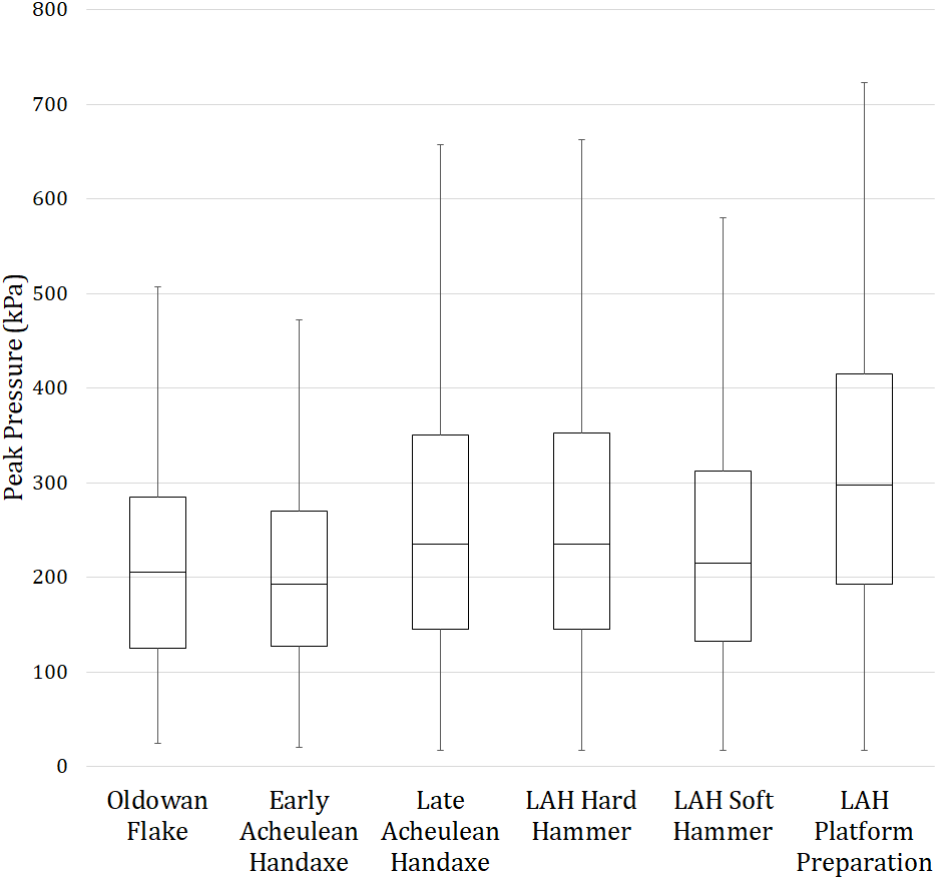
Boxplots depicting peak pressure data. The nine knappers during the three types of stone tool production strategies (*n* = 506, 777, and 1,503 for the Oldowan flake, EAH, and LAH data, respectively) and the three mass removal strategies utilised in the late Acheulean handaxe reduction sequence (*n* = 617, 617, and 269 for the hard hammer, soft hammer, and platform preparation data, respectively) are shown.

Descriptive data for the pressure values used in each analysis are detailed in Tables 1–5. Between the three types of tool production sequence there were substantially more mass removal events when producing LAHs (*n* = 1,503), relative to flakes and EAHs (*n* = 506 and 777 respectively; Table 1). Around twice as many flake removals were required during the production of LAHs relative to EAHs. Mean, summed peak pressure records across the non-dominant hand during the production of LAHs were also greater than the flake and EAH sequences by ∼50 kPa (Table 1; Fig. 7). The Friedman test did not reveal significant differences between median pressures used in the three types of reduction (*p* = 0.22138) and so post-hoc tests were not conducted. Although the production of ‘Late Acheulean Handaxes’ required greater mean pressures to be exerted and resisted by the non-dominant hand across all data collected, compared to the production of Oldowan flake tools or ‘early Acheulean handaxes’, these differences were not significant.

**Table 1:** Descriptive data outlining the differences in combined peak pressure data recorded on the non-dominant hand during flake and core, ‘early Acheulean handaxe’, and ‘late Acheulean handaxe’ stone tool production sequences.

All manual behaviours combined	*n*	Mean (kPa)	Median (kPa)	S.D. (kPa)	Min (kPa)	Max (kPa)
Flake	506	214.3	205	114.2	25	722.5
EAH	777	203.5	192.5	104.5	20	617.5
LAH	1,503	261.8	235	155.8	17.5	930

**Table 2:** Data describing combined peak pressure differences between successful and unsuccessful flake removals within the three types of stone tool production sequence examined.

Flake removal success		*n*	Mean (kPa)	Median (kPa)	S.D. (kPa)	Min (kPa)	Max (kPa)
Flake	Successful	393	210.7	205	111.5	25	722.5
	Unsuccessful	113	227.1	205	122.8	25	525
EAH	Successful	636	200.5	188.8	103.7	22.5	617.5
	Unsuccessful	141	217.5	212.5	107.1	20	535
LAH	Successful	991	246.4	220	151.1	17.5	930
	Unsuccessful	243	256.5	225	137.7	25	705

**Table 3:** Differences in combined peak pressure recorded on the non-dominant hand of the nine knappers during flake, EAH, and LAH reduction sequences, dependent on whether hand or leg core support strategies were used.

Core support strategies		*n*	Mean (kPa)	Median (kPa)	S.D. (kPa)	Min (kPa)	Max (kPa)
Flake	Hand	315	220.7	217.5	114.8	25	577.5
	Leg	191	203.8	190	112.6	25	722.5
EAH	Hand	367	187	180	85.3	25	465
	Leg	410	218.3	205	117.2	20	617.5
LAH	Hand	503	291	260	152.3	20	802.5
	Leg	1,000	247.2	200	155.5	17.5	930

**Table 4:** Data describing the combined peak pressure recorded on the non-dominant hand of knappers while producing ‘late Acheulean handaxes’ (see definition provided in the main text), dependent on the type of technique used to remove mass from the core.

Mass removal strategy		*n*	Mean (kPa)	Median (kPa)	S.D. (kPa)	Min (kPa)	Max (kPa)
LAH	Hard hammer	617	264.9	235	159.5	17.5	930
	Soft hammer	617	231.8	215	134.8	17.5	785
	Platform preparation	269	323.5	297.5	172.8	17.5	802.5

**Table 5:** Descriptive data for the combined peak pressure records used in the four regression analyses with flake removal numbers.

Reduction stage (flake removal number)	*n*	Mean (kPa)	S.D. (kPa)	Min (kPa)	Max (kPa)
Flake	506	214.3	114.2	25	722.5
EAH	777	203.5	104.5	20	617.5
LAH	1,234	248.4	148.5	17.5	930
LAH Platform Preparation	269	323.5	172.8	17.5	802.5

Ratios of successful to unsuccessful flake removals varied only slightly between the three reduction strategies (ranging between 7:2 and 9:2) (Table 2). In each strategy, successful flake removals reported pressure values ∼10–15 kPa below unsuccessful removals (Table 2). Mann–Whitney *U* tests identified that these differences were not significant in any of the three sequences (*p* = .069–.249). In turn, the success of flake removals does not seem to be a consequence of variation in pressure exerted by the non-dominant hand during stone tool production, although there is consistency in successful flake removal recording marginally lower pressure values.

Core support strategies varied between the leg and hand in all three reductions. In terms of data frequency there is a split between flake production, which reports greater use of hand support, the EAH reductions which are broadly equal between the two, and the LAH reductions where there were clear preferences for cores being supported by the leg (Table 3). While no significant pressure difference is recorded between the hand and leg support techniques during flake production (*p* = .060), both of the handaxe sequences report significant differences (*p* =   ≤ .001; Table 3). However, during the EAH reduction greater pressure values are reported during leg support while LAHs report greater values during hand support (Table 3). The technique used to support a stone core therefore appears related to the pressures required to secure it during flake removals and platform preparation events, however, differences appear dependent on the type of tool being produced.

It was only possible to compare hard hammer flake removals, soft hammer flake removals, and platform preparation events during the LAH reduction sequence. Across the nine participants there were equal numbers of hard and soft hammer flake removals (*n* = 617 for each removal type), suggesting that both types of percussor are equally important during LAH production sequences (Table 4). There were, however, 4.6 times as many flake removals relative to platform preparation events, indicating that only ∼one in five flakes required its platform to be prepared prior to its removal. When only soft hammer percussion is considered, where platform preparation may more normally be expected, every other flake was removed without its platform being prepared (i.e., one in two flakes had its platform prepared). Soft hammer percussion returned, on average, the lowest peak pressure records across the hand (Table 4; Fig. 7). Hard hammer percussion required an additional 33 kPa of pressure to be exerted and resisted by the non-dominant hand. An additional 59 and 92 kPa were recorded, on average, across the non-dominant hand of knappers during platform preparation events compared to hard and soft hammer percussion, respectively (Table 4; Fig. 7). The Friedman test between normalised median pressures used in the three types of mass removal was significant (*p* = .0001). Subsequent pairwise Wilxcoxon signed rank tests indicated that platform preparation events required significantly more pressure than both hard (*p* = 0.0002) and soft hammer (*p* = .0043) removals, while there was no significant difference between the latter two mass removal types. Platform preparation events do, therefore, appear to require significantly greater pressure to be exerted and resisted by the non-dominant hand compared to both hard and soft hammer flake removals.

**Table 6:** Regressions between both the original and ‘standardised’ flake removal or platform preparation sequence event numbers and combined peak pressures experienced on the non-dominant hand.

	*p*	*R*^2^
**Original flake removal numbers**		
Flake	.0001	−.029
EAH	<.0001	−.030
LAH	<.0001	.066
LAH platform preparation	<.0001	.416
**Standardised flake removal numbers**		
Flake	.0007	−.023
EAH	<.0001	−.090
LAH	.0035	.007
LAH platform preparation	.0078	.026

**Figure 8:**
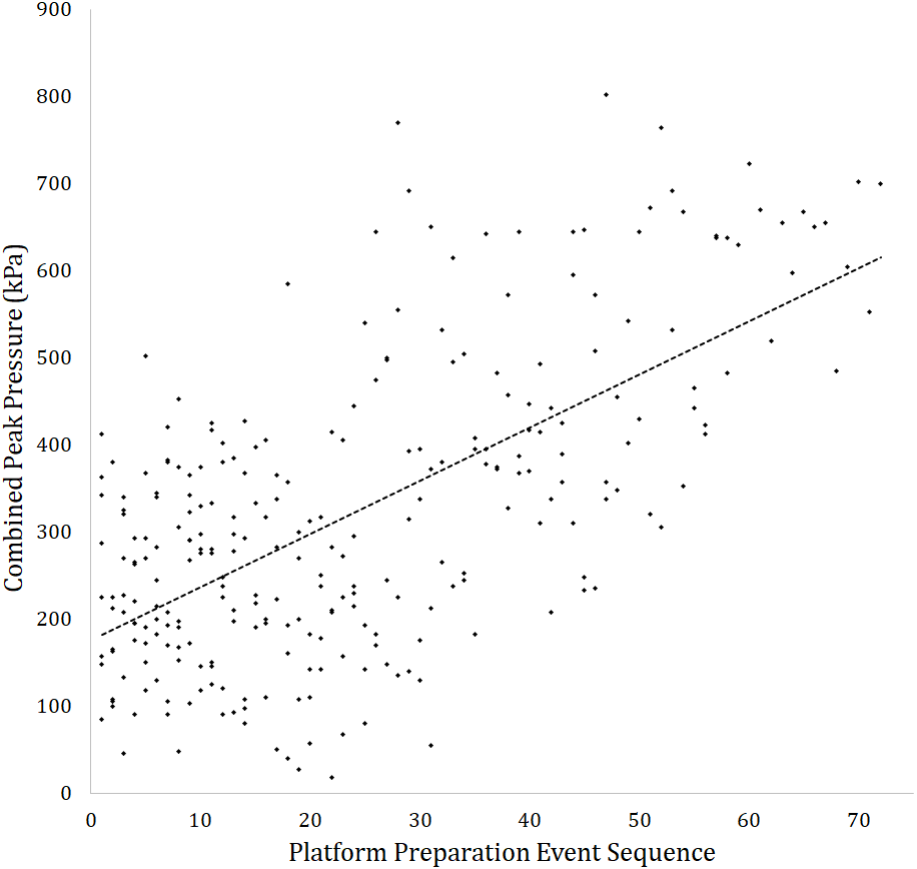
Linear regression between the combined peak pressures recorded on the non-dominant hand of knappers during platform preparation events (grinding and trimming) and the sequence of that platform preparation events (R2 = .416). All data are from LAH reduction sequences only.

The LAH data values used during the regression analyses were, on average, greater than both the flake and EAH reductions (by 34 and 45 kPa, respectively) despite the absence of platform preparation events (Table 5), demonstrating that even in the absence of this uniquely late Acheulean behaviour, the production of LAH forms requires greater manual pressures. Of the eight linear regressions undertaken all identified significant relationships between flake removal sequence numbers and manual pressure (Table 6). Flake and EAH reduction sequences displayed negative relationships, whereby pressure decreased as reduction sequences progressed. LAH sequences and LAH platform preparation events displayed positive relationships, indicating that later mass removal events required greater manual pressures (Table 6). In all but one instance *R*^2^values were ≤.090, indicating that limited (≤ 9%) pressure variation could be attributed to a core’s stage of reduction. The single exception was the regression between LAH platform preparation sequence numbers and their respective pressure values, where 42% of the observed pressure variation could be attributed to the stage of a handaxe’s production (Table 6; Fig. 8). This indicates that as late Acheulean handaxes progress further through production sequences (i.e., as they become smaller, increasingly shaped and thin relative to their thickness) the pressure required to stabilise them during platform preparation events increases significantly. The fact that this relationship is not similarly repeated in the normalised flake removal sequence numbers indicates that this relationship is unlikely to be driven by how close a handaxe is to being considered finished by the knapper, but by how long the sequence goes on for, how many flakes have been removed, and how ‘refined’ a biface becomes.

## Discussion

The present work investigates the origin of technological innovation during the Lower Palaeolithic from a biomechanical and evolutionary perspective, and asks whether the onset of new stone tool forms and production techniques may have been restricted by hominin manual capabilities. Our results demonstrate that although later Acheulean handaxes (LAH) required the exertion and resistance of greater manual pressure during their production relative to either Oldowan flake and core tools or early ‘rougher’ Acheulean handaxes (EAH) (by an average of 22% and 29%, respectively, when all data were considered), these differences were not found to be significant and may have been driven by a few individuals. It is, therefore, not possible to state that manual pressure requirements during flake detachments vary significantly between the three tools examined here.

However, the preparation of LAH flake platforms, through retouching and edge grinding, elicited the greatest loads in this study. Indeed, the action of preparing a flake’s platform prior to its removal required significantly (22–40%) more pressure than soft or hard hammer flake removals in the same reduction sequences (Table 4; Fig. 7). Compared to Oldowan or EAH flake removals, mean pressures are 55–59% (>110 kPa) greater during LAH platform preparation events (Tables 1 and 4; Fig. 7). This result suggests that platform preparation techniques may only have been possible for hominins capable of performing particularly forceful precision grips. These grips would have required greater force than those needed for earlier stone tool types. Arguably, only once hominins evolved enhanced manipulative capabilities in response to selective pressure exerted by earlier manual behaviours, would the innovation of later Acheulean handaxe forms, produced using the preparation of flake platforms, have been possible. Such behaviours include flake tool use, hammerstone use, and Oldowan/EAH core manipulation (Marzke, 1997; Marzke, 2013; Kivell, 2015; Key & Dunmore, 2015; Williams-Hatala et al., 2018). As highlighted by Tocheri et al. (2008), fossil hand anatomy indicates the continued derivation of hominin manual capabilities subsequent to the onset of the Acheulean, which may have facilitated the forceful grips used for securing the core during platform preparation events, required for LAH production.

During platform preparation events edges are modified either via the removal of very small flakes when isolating as well as reshaping platforms or altering their angles, or they can be reduced, bevelled, reshaped and isolated through forceful grinding actions. In each case, these actions require the precise but forceful application of stone or antler against the handaxe’s edge. In turn, it is essential for handaxes to remain stable throughout this process so that the percussor or grinding stone is applied only to the specific area being shaped (for refined bifaces flake platforms are often <10 × 5 mm). Regarding small flake removals, it is the highly precise nature of the removals that necessitates a particularly firm and steady grip on the handaxe.

The act of grinding a handaxe’s edge in preparation for a flake removal, however, also requires the input of substantial and prolonged forces through an abrasive stone onto the biface’s edge. In addition to their extended duration, it is likely that the dominant hand at times creates forces in excess of those observed during flake detachments. Certainly, during edge grinding the palm contributes substantially to the loads transferred onto a core, something that is impossible during most hammerstone strikes (and therefore flake detachments). While previous biomechanical studies of the dominant hand have tended to overlook edge grinding events (although see: Marzke & Shackley, 1986), and thus these claims cannot yet be substantiated, our pressure data clearly identifies a requirement to oppose substantial reaction forces during platform preparation events. More specifically, these pressures are significantly greater than those observed during flake removals.

When LAHs are secured during platform preparation events up to 42% of the pressure variation recorded here can be attributed to the stage of a handaxe’s production, demonstrating proportionally greater force is required to prepare platforms for progressively refined flake removals (Fig. 8). This relationship cannot be straightforwardly attributed to participant fatigue, as platform preparation events and flake removals were undertaken throughout reductions and no fatiguing was reported or observed. Rather the form of the handaxe (core) being supported and secured is likely responsible for this result.

As any reduction sequence progresses, cores become smaller (Clarkson, 2013; Douglass et al., 2018) and handaxe size has been experimentally demonstrated to have a strong negative relationship with reduction intensity (Shipton & Clarkson, 2015a; Shipton & Clarkson, 2015b). Marzke & Shackley (1986) found that as reduction sequences progress the thumb and distal aspects of the fingers are increasingly used in isolation when gripping the core to secure it against hammerstone strikes (see also: Pouydebat et al., 2009). As a corollary, both the greater surface area of the palm and the most ulnar digits (fourth and fifth) are used progressively less (Marzke et al., 1998), which concentrates manual forces on the radial three digits. This concentration of force thereby increases the pressures required to produce, typically smaller, LAH’s.

The stage of a handaxe’s reduction also has potential to impact its volumetric distribution and shape (Crompton & Gowlett, 1993). Archer & Braun (2010) demonstrated that as reduction sequences progress, a handaxe’s centre of mass moves first to the centre of the tool and subsequent thinning flakes move it to the tool’s base. As highlighted by Faisal et al. (2010), this results in an increased requirement to properly secure and brace the tool during flake removals and platform preparation events. Certainly, during the latter stages of LAH production there is increased risk that a biface will break (i.e., fracture in an unintended way) when flake removals are instigated. This may be through the intended fracture ‘diving’ through the biface when searching for the route of least resistance, or by reaction forces propagating through the tool and creating stress enough to fracture in additional locations (often the tip). In both cases, the principle means for a knapper to prevent these mistakes (other than choosing suitable flakes to remove) is by forcefully bracing the length of the biface. While most effect sizes were small, the other regression analyses support this idea as flake and EAH regressions display negative relationships with pressure but LAH sequences show positive relationships. During flake and EAH reductions the reducing core mass requires less support and stabilisation, resulting in lower manual pressure. While this may also characterise early stages of LAH production, as sequences progress, pressure increases substantially. It is likely that the production of bifacially flaked tools with even lower thickness to width ratios, such as Solutrean or Clovis points (e.g., Smallwood, 2010; Eren et al., 2013), would require even greater pressures.

### Wider implications

Stout and colleagues (Stout et al., 2008; Stout et al., 2015), and more recently Muller, Clarkson & Shipton (2017), have demonstrated that Acheulean handaxe production requires increased visuomotor coordination, hierarchical organisation and is more cognitively demanding than Oldowan flake tool production. Wynn (2002) and Muller, Clarkson & Shipton (2017) further suggest late Acheulean handaxe production sequences to be more complex than those required for early Acheulean handaxes. When combined with the present study, the production of later Acheulean handaxes could, therefore, also be considered a biomechanically *and* cognitively more demanding behaviour than earlier types of stone tool production. Although earlier research hinted at how manually demanding later handaxes were to produce (e.g., Krantz, 1960; Marzke & Shackley, 1986), it is only now that there are empirical data in support of this conclusion. Earlier work by Faisal et al. (2010) investigated the manipulative complexity (variation) of Oldowan and late Acheulean handaxe reduction strategies did not find any notable differences in digit joint or abduction angles. Our platform preparation results may, at first, appear in contrast to those reported by Faisal et al. (2010) insofar as we did find significant manual differences between Oldowan flake and late Acheulean handaxe production. Each study, however, investigates or infers a distinct biomechanical element of stone tool production. That is, the manual demands associated with joint angle *complexity* are not tantamount to demands associated with *loading* levels. So while the complexity of these behaviours have not been demonstrated to be different (Faisal et al., 2010), the production of late Acheulean is still a more demanding manual behaviour, but only in terms of the manual pressure levels resisted and exerted.

Key and colleagues (Key et al., 2017; Key & Lycett, in press) have argued that the production of large flakes (e.g., >10 cm) via hard hammer percussion and the effective use of handaxes, which are both characteristic features of early Acheulean tool assemblages (De la Torre & Mora, 2014), required manual biomechanical prerequisites prior to their widespread adoption by hominin populations. The present study suggests that the removal of bifacial flakes from a core when shaping an EAH is no more demanding, in terms of loading on the non-dominant hand, than the removal of flakes from a core during more straightforward Oldowan core reduction strategies. So, while there may be other manual prerequisites prior to the adoption of early Acheulean technologies (Key et al., 2017; Key & Lycett, in press), the loads required to secure cores do not appear to be one. As far as the present study demonstrates, we can attribute the specific technological development of core shaping through bifacial flake removals (*n.b.* not large flake production or the effective use of these tools (Key et al., 2017; Key & Lycett, in press)) to be more likely linked to changes in hominin cognitive, cultural, or linguistic capabilities (Wynn, 2002; Uomini & Meyer, 2013; Stout et al., 2014; Morgan et al., 2015; Schillinger, Mesoudi & Lycett, 2015; Stout et al., 2015; Lycett et al., 2016), or increased functional and ecological demands for large tools with scalloped cutting edges (Jones, 1980; Key & Lycett, 2017a; Key & Lycett, 2017b; Wynn & Gowlett, 2018), than biomechanical restrictions.

### Further technological considerations

Our finding that flaking success cannot be attributed to pressure levels when securing cores demonstrates that, for skilled knappers at least, other factors are more important in determining flake detachment success. We are not suggesting that a secure and forceful grip on stone cores is not essential to the successful removal of flakes. Neither do we mean to imply that the loads required to secure a core do not change in response to different morphological or technological aspects of a tool production sequence (e.g., flake and core size, platform angle, percussor type). The high but variable loads exhibited here attest to these requirements, as do results reported in previous studies (Marzke et al., 1998; Key & Dunmore, 2015). Rather, our results demonstrate that the visuomotor control of skilled flint knappers during stone tool production is such that they can appropriately judge manual pressure requirements during flake detachments with equal success across the three types of reduction strategies examined here. Although, of course, there is potential for considerable variation in appropriate or necessary pressure outputs (*cf.* Rein, Nonaka & Bril, 2014; Key et al., 2017). Given the experience of the knappers used in this study, indications of advanced motor-skills during flake detachments are not surprising (Nonaka, Bril & Rein, 2010). Nonetheless, it is interesting that the success of flake removals by skilled flintknappers cannot be attributed to the use of higher or lower than required loading through the non-dominant, core securing, hand. It is beyond the scope of the present study to comment on whether the success of flake removals by novice knappers can, at least in part (Nonaka, Bril & Rein, 2010; Stout et al., 2015), be attributed to an inability to appropriately judge the loads required to secure a core. Interestingly, the ratio of ∼4:1 successful to unsuccessful flake removals (991 successful and 243 unsuccessful flake) across the LAH reductions was repeated when only flake removals performed immediately after platform preparation events were considered (160 successful and 38 unsuccessful flaking attempts). Indicating that, at least for expert knappers, the preparation of flake platforms does not increase the success of flake removals.

Both handaxe reduction sequences demonstrated significant pressure differences between the hand and leg core support strategies. The EAHs required greater values during the leg support technique while the LAH required greater values during the hand condition. The cause of this difference may relate to the disproportionate use of each support strategy at different stages of a reduction sequence, changes in grip choice and pressure requirements as reductions progress, and the inclusion of platform preparation data in the LAH reduction. All reduction types used the leg support strategy more frequently during the earlier stages of a reduction sequence. This was likely because the most comfortable way to support a particularly heavy core’s weight was by using the leg, with the hand chiefly being used to stabilise the core against hammerstone strikes. As sequences progressed cores became smaller, meaning that it was easier to support and secure cores using only the hand. A shift to the more frequent use of a hand support strategy also coincided with the already discussed need for greater pressure as cores become more ‘refined’ during platform preparation events. The greater duration of LAH reductions would have created increased opportunity for high loading. The greater frequency of the leg support technique during handaxe reductions, but most notably the LAH sequence, is likely due to the greater stability of this technique. As handaxes become thinner relative to their thickness they are more likely to break during flake removals. The use of the leg as a supportive structure allows for greater areas of the biface to be firmly secured by the body, decreasing the likelihood of it breaking during flake removals. Such comprehensive support is rarely required during ‘simple’ flake production strategies, hence, the leg support technique is more frequently being used in early stages of flake production.

Although soft hammer percussion was used more frequently during the later stages of LAH sequences, this percussive technique did not contribute to greater pressures values during the hand support strategy, nor the greater pressures recorded in the later stages of LAH reduction sequences. Indeed, soft hammer percussion required similar loads to hard hammer percussion. This is despite soft hammers being more frequently used to remove smaller flakes (in terms of mass, if not length), in turn requiring lower impact forces (Dibble & Rezek, 2009) and creating lower reaction forces to be resisted. Irrespective of the cause, our data indicates that the seemingly delayed onset and adoption of soft hammer percussion during the later stages of the Lower Palaeolithic (Copeland, 1991; Schick & Clark, 2003; Stout et al., 2014) cannot be attributed to biomechanical limitations in the non-dominant hand of hominins.

### Limitations

It is important to note that the pressures recorded here are not likely representative of the total forces exerted and resisted by the non-dominant hand during stone tool production. As past research demonstrates (Marzke & Shackley, 1986; Key & Dunmore, 2015), the palm plays an important role in supporting cores during flake removals (e.g., Figs. 4A and 4B) and the sensor array used here did not take this into account. It is hard to say whether the inclusion of palmar pressure data would have altered any of the present results, but indications of an increased reliance on distal aspect of digits during later stages of reductions highlights the need for future research to take this into consideration. Further, although the number of behaviours analysed here is, as far as we know, the largest yet recorded during an investigation into stone tool related manual loading (*n* = 2786), only nine skilled flintknappers were able to take part in the study. In turn, and as already discussed, there is potential for our data to be significantly influenced by a few individuals. This includes differences caused by variable grinding and core securing techniques learnt as each knappers first developed their knapping capabilities, and the possibility of an individual not providing ‘natural’ nor repeatable data on the specific day data were collected. Although there does not appear to be any indication that this has happened, we cannot rule this possibility out in totality. Hence, we would welcome the publication of similar studies in the future that are able to examine increased numbers of knappers.

## Conclusion

The Lower Palaeolithic artefact record represents the largest and most detailed record of the minimum technological capabilities of hominins during the Plio-Pleistocene. As such the Oldowan and Acheulean periods track significant shifts in the behaviour of hominins, which have been investigated in terms of cognition, social transmission, environmental factors and others. Here we investigate these transitions from a biomechanical perspective, as inferred from manual loading data. Our results demonstrate that the digital pressures required to forcefully secure later Acheulean handaxes during their production are not significantly greater than those required when knapping earlier Acheulean handaxe forms or Oldowan flakes. However, the novel LAH associated behaviour of preparing flake platforms would have required significantly stronger grips in the non-dominant hand compared to earlier stone tool production behaviours. Therefore, we contend that the behavioural shift marked by the onset of platform preparation behaviours, as observed in later Acheulean handaxe forms, may be intrinsically linked to the biomechanical capabilities of hominins, among other factors, in a co-evolutionary manner.

##  Supplemental Information

10.7717/peerj.5399/supp-1 Data S1 Experimental data

10.7717/peerj.5399/supp-2 File S1 R script for uniform increment subsampling

## Additional Information and Declarations

### Data Availability

The following information was supplied regarding data availability: The raw data are provided in Data S1.
